# The Reaffirmation of Self? Narrative Inquiry for Researching Violence Against Women and Stigma

**DOI:** 10.1177/10778012211024269

**Published:** 2021-08-23

**Authors:** Carol Ballantine

**Affiliations:** 1 Centre for Global Women’s Studies, School of Politics and Sociology, 8799 NUI Galway, Dublin, Ireland

**Keywords:** stigma, violence against women, narrative inquiry, race, refugees

## Abstract

Stigma presents specific ethical and epistemological problems for qualitative researchers of violence against women. Narrative research methods promise to enable ethical research on violence while still offering deep insight into stigmatized topics. This article describes narrative methods used in six focus group discussions and four in-depth interviews with victim-survivors of violence against women, all African migrant women living in Ireland. The article connects narrative and stigma in research with the social lives of participants. It concludes with specific recommendations for creative uses of narrative inquiry to explore stigmatized themes, noting that stigma can never be entirely removed from the research encounter.

In *Stigma: The Machinery of Inequality*, Professor Imogen Tyler (2020) describes her own experience of the anthropological gaze. Tyler recalls attending a dinner with some academics, at which an anthropology PhD student gave a slideshow from her fieldwork in Papua New Guinea. On hearing the name of the small Lancashire village where Tyler grew up, the anthropologist exclaimed: “I don't believe it, my PhD supervisor did his fieldwork there!” Tyler goes on to describe her dreams that night, in which she finds herself and her community projected on the anthropologist's wall:

The dinner guests don't recognise that I am in the images. They talk about the culture of the rural community; they share comments, offer analysis, make judgements. It is impossible for them to imagine that the object of the fieldwork might be right there with them in the room and on the wall at the same time, in the same space and historical time. Served up on the wall to be consumed at an academic dinner party. I shrink into the chair. I make myself as small as possible. I want to disappear. … I am overwhelmed with shame. I radiate shame. (Tyler, 2020, p. 230)

Tyler's searing account lays bare the challenge that confronts the researcher of stigma: How are we to understand stigma without ourselves reproducing it? Since violence against women is a subject heavily bound by multiple stigmas, this challenge is especially pertinent for researchers of such violence. Narrative research methods, particularly creative and participatory methods, promise to restore agency to the stigmatized individual and even empower her to take control of her depiction in the world. In this article, I outline how I used narrative methods in a research study on stigma and gender-based violence with African migrant women in Ireland, and reflect on the uses and limitations of narrative inquiry for both understanding stigma and challenging it.

The first section of this article introduces the methodological challenge of researching stigma and violence and explores the possibilities of narrative for overcoming some of these challenges. Research practice can produce stigma—as in the example at the beginning of this article—and stigma and associated shame can inhibit good research. Given its concern with restoring agency to excluded research subjects, narrative inquiry presents the opportunity to conduct research ethically, and go beyond the epistemological black box of stigma, an opportunity this article explores in detail.

The second section of the article outlines the qualitative research methods I used in a mixed methods research study, including an in-depth qualitative study of life experiences of violence with African migrant women living in Ireland. I discuss six participatory focus group discussions (FGDs) and four in-depth interviews (IDIs), describing how these were structured as narrative research with examples of the actions I took to keep narrative at the center of the research approach. Before outlining the research procedure, I introduce the interview participants by name with a short description of their overall testimony. A significant aspect of navigating stigma for the interviewees was in the ways they told their stories, uniting the research methods and research findings.

In the third section, I discuss the application of narrative inquiry in the context of my research. For interview participants, self-identified victim-survivors of violence, the methods used were successful in giving a sense of agency and autonomy. Narrative also proved effective to stimulate open and insightful discussion in both FGDs and IDIs, yielding valuable knowledge related to the navigation of stigmatized lives. In a small-scale study, I note that interview participants were highly motivated to narrate their own experiences of violence and oppression. Drawing on four different interviews, I describe a clear connection between narrative within the interview and outside of it, which researchers of violence should pay close attention to. However, narrative is also an important way that stigma is communicated and (re)produced, and this was apparent in this study. I discuss the representation of stigmatizing beliefs in the study, and identify stigma as an important factor in shaping narrative possibilities for participants in the study.

The article concludes that the use of creative, feminist narrative methods enabled this study to explore stigma without endorsing it, and describes specific actions that facilitated this. It ends with recommendations for qualitative researchers of violence against women and stigma, and a caution to researchers not to be naive about the possibilities of eliminating stigma or redressing structural power imbalances through narrative methods.

## Understanding Stigma and Violence

The study of stigma poses a particular methodological problem. By zoning in on stigma as a topic, researchers—particularly those unaffected by the stigma themselves—may perpetuate unsubstantiated assumptions (Link & Phelan, 2001), thus recreating the very phenomenon that, through study, we aim to eliminate.

I follow Link and Phelan (2001) in defining stigma as a feature of social relationships, the process whereby a particular attribute (e.g., a behavior, profession, medical status, or physical condition) is labelled as socially significant, and stigmatized individuals are subjected to status loss as a result. In line with Link and Phelan (2014) and other researchers of the sociology of stigma (e.g., Parker & Aggleton, 2003; Tyler, 2020), I note that stigma always occurs in a particular power situation: stigma is itself an exercise of power (Link & Phelan, 2014; Tyler, 2020). Stigma has both social and individual components; in this sense, it is co-produced by individuals in social settings (Goffman, 1963). Its most insidious impacts are often its impacts on the sense of self (Goffman, 1963; Link & Phelan, 2014; Murray et al., 2015). An important impact of stigma is that it can lead to the emotion of shame, a painful negative evaluation of the global self, or the whole identity (Manion, 2003).

Stigma poses similar ethical and methodological challenges to those related to “sensitive topics” (Fontes, 2004). There is a distinction in that while sensitive topics are those which might cause an individual distress or discomfort, stigmatized ones are those which additionally threaten the research participant with social devaluation and status loss, potentially a catastrophic outcome, particularly in small, socially isolated refugee communities (Tankink, 2013).

In this article, I follow the definition of violence against women used in the UN Declaration on Elimination of Violence Against Women:

any act of gender-based violence that results in, or is likely to result in, physical, sexual or psychological harm or suffering to women, including threats of such acts, coercion or arbitrary deprivation of liberty, whether occurring in public or private life. (UN General Assembly, 1993)

It is a truism of violence against women research and practice that victims and survivors are stigmatized, although stigma and associated shame are more often named as barriers to research than as topics for research in themselves (notable exceptions include Thaggard & Montayre, 2019; Tonsing & Barn, 2017; Murray et al., 2015).

Throughout the world, including in most African countries from which participants in this study originated, women are affected by patriarchal stigma, which targets any sort of deviance from culturally prescribed normative femininity, including single parenthood, divorce and separation, and failure to perform wifely duties (e.g., Kalunta-Crumpton, 2016; McCleary Sills et al., 2016; Ting & Panchanadeswaran, 2009). In this sense, both violence and stigma are equal and mutually reinforcing parts of the system of patriarchal control of women. Gender-based violence against women itself is also specifically stigmatized. For example, in the case of nonpartner sexual violence, victims are frequently labelled as “spoiled” or “dirty” (e.g., Barnett et al., 2016). In the case of intimate partner violence (IPV), migrant African women in the United States have described being socially judged for their poor choice of partner (Ting & Panchanadeswaran, 2009) or their failure to leave a violent partner (Kalunta Crumpton, 2016), while a study in Ghana (Alvarado et al., 2019) demonstrated that women victimized for IPV would not be chosen as community leaders owing to their “impaired judgement” for ending up with a violent partner.

The research being presented in this article was conducted with migrant African women living in Ireland. The concern with stigma is elevated, because alongside patriarchal and potentially violence stigma, this group is subject to many other social stigmas. I discuss this further in the description of the study below.

## Researching Stigma and Violence

Stigma complicates the complicated business of doing research into violence against women, because it promotes silences and causes harm. Stigma poses two distinct challenges to research on such violence. The first of these challenges is ethical: How are we to carry out research without exposing the research participants to the harms of stigmatization? The second is epistemological: Stigma and shame are difficult to research since they often hide detail and nuance behind stereotypes and cause their subjects to conceal the information that the researcher seeks to explore. This research challenge is often referenced but less frequently engaged in violence literature, as in this comment on the state of the global research: “Obtaining accurate figures on the prevalence of sexual assault by non-partners … is particularly difficult because of the stigma attached to it” (Ellsberg, 2006, p. 1).

Frequently, stigma is heavily present in the cultural setting of the research exchange, yet difficult to address directly. In the WHO guidebook on researching violence against women, Ellsberg and Heise (2005) note that focus groups are not an appropriate setting for disclosure of individual or personal details. Tankink (2013) provides the instructive example of focus group research with refugee women from South Sudan living in the Netherlands. For this cohort, talking about sexual violence with their community was considered a harm equivalent to the violence itself, since disclosing the stigmatized status of the victim-survivor would amount to severing vital social connections. Tankink's research participants were able to disclose their experiences of sexual violence in individual interviews with her as an outsider interviewer, but not in focus groups. Nor are these challenges entirely escaped in interview encounters. Rance et al. (2017, p. 2224) describe a narrative interview with “Jimmy” (a pseudonym) on the subject of drug injection and Hepatitis C. They show how their stigmatized interview subject, discussing his stigmatized identity as an injecting drug user, was “required to negotiate the stigma that has been invited to take centre stage in the research.” Jimmy's narrative is inescapably shaped by the fact of being stigmatized, even when the researchers seek to eliminate stigma in the interview. The story he tells is infused with the stigma he navigates in daily social encounters.

Researching stigma is necessary and important. However, the ethical challenges to the individuals in the research encounter and to the wider construction of knowledge are significant. These issues call for a constant focus on methods which address both violence against women and stigma as research topics.

In order to move beyond detailing stigmatizing beliefs and attitudes towards investigating how individuals navigate and accommodate stigma, nuanced and ethical methods are necessary. The reproduction of stigma in research may be unavoidable, but a question posed by research on gendered violence in highly patriarchal settings is whether the research encounter might at least enable alternative interpretations of the meanings of violence? This is proposed by both Tankink (2013), considering refugee women's experiences of sexual violence; and Polletta (2009), in an essay which considers new plots in stories about battering. These questions open the door to the uses of narrative in research on violence against women and stigma.

## A Narrative Study of Violence and Stigma

Narrative inquiry is concerned with meaning making at the human level: A narrative is an individual's own account of something. Narration, says Hannah Arendt, “reveals the meaning without committing the error of defining it” (quoted in Cavarero, 2000, p. 3). Narrative, of course, is most commonly seen as another word for story: the way in which individuals relate experiences, and in so doing make meaning of them (Elliott, 2005).

Narrative research is considered methodologically valuable for the study of disempowered and marginalized people, particularly in refugee studies (Eastmond, 2007) and research on violence against women (Boonzaier & van Schalwyck, 2011). As an approach, narrative aims to privilege the voice of the research participant and place their experiences in the wider context (Eastmond, 2007), allowing research to expose the complexities and contradictions of real lives. Against the universalizing tendencies of stigmas, narrative can present individuals in all their “unrepeatable uniqueness” (Cavarero, 2000).

As individuals, we live storied lives and narrate constantly. Thus, research is just one of hundreds of spaces in which we do narrative. My work with narrative draws on feminist participatory methodologies which aim to democratize knowledge by diminishing, as much as possible, the power imbalance between researchers and the groups of people at the center of inquiry (Waight, 2020). The study described in this article is a feminist, creative, and participatory narrative study. In describing my research as feminist, I mean that I am concerned with gendered hierarchies in society, and I actively seek to identify the impacts of such hierarchies. My research is socially engaged and aims to be accountable to its participants (Harding & Norberg, 2005). I work to reduce, inasmuch as I can, the power differences between myself and my participants (Harding & Norberg, 2005).

Applying a narrative approach to research has both an inherent and an instrumental value. The process has the inherent value of claiming and owning one's own story (Eastmond, 2007). Survivors of trauma, argues Brison (2002), sometimes need not alone tell their story in order to survive, but for their story to be heard or witnessed by others.

The instrumental value of narrating lies in the possibility that by presenting contextualized, nuanced details of the storied life, the perceptions of others relating to an individual, group, or issue might change. Eastmond (2007) shares the example of a research project with survivors of Chile's military regime, for whom narrative research offered the promise of being publicly acknowledged and believed. Closer to home for me (and my research participants) was the experience of Ireland's referendum campaign to repeal the eighth amendment to the Irish constitution, which made abortion illegal in all circumstances. It was the stories of hundreds of women who had abortion-related experiences under the eighth amendment that changed the conversation in Ireland. The crowd-sourced Facebook page In Her Shoes—later a book—had a significant impact on the public conversation (Enright et al., 2020). Such is the possible instrumental power of narrative.

Narrative researchers acknowledge—indeed, embrace—the fact that data are co-constructed in the interaction between researcher and participants (e.g., Boonzaier & van Schalwyk, 2011). This calls on researchers to be aware of their own part in the creation of the narrative as it emerges in the context of interviews and other encounters: The presence and actions of the researcher shape and construct the narrative that is created, alongside many other factors and conditions, both known and unknown to the researcher. Among these factors are social stigmas, which are inevitably present in the research encounter even when the researcher works to eliminate them (Rance et al., 2017). Indeed, without dedicated attention to the subject, researchers can invite participants into situations and spaces in which stigma is reproduced and research participants are stigmatized.

Narrative research methods certainly offer an ethically sensitive approach to the tricky task of researching stigma and violence. Such methods are explicitly opposed to “othering” of research participants, and set out to promote agency. The invocation of stigma in research encounters, especially group encounters, might be addressed by facilitating the emergence of alternative, nonstigmatizing narratives (Tankink, 2013). Participatory, creative, and feminist narrative research methods may additionally enable research to get closer to the multiple truths of stigmatized lives, and past the stereotypes and bland assertions which can characterize research literature on the topic of stigma and violence against women. In spite of all this, stigma still impacts research and requires careful consideration. This is the methodological backdrop which informed the current study design and which I discuss below.

## Creating Spaces for Nonstigmatizing Narratives

### The Research Study

My PhD investigated the long-term social impacts of violence against women on African migrant women living in Ireland. Using a framework of the continuum of violence and oppression (Kanyeredzi, 2018; Kelly, 1988), the research investigated the role that stigma plays in mediating the impacts of violence on women's space for action (Kelly, 2003). There is a paucity of qualitative and quantitative data related to violence against women in Ireland generally, and especially as it affects specific subpopulations such as African migrant women in Ireland. African women represent an understudied population within the overall underexamined area of gender-based violence in Ireland and, additionally, a population that offers a rich and underexplored space for examining questions related to gender, violence, and stigma. Stigma is relevant to this group, who are raced and gendered as other, resulting in quantifiable discrimination and disadvantage in, for example, employment (Joseph, 2018), housing (Michael, 2015), and social welfare (O’Brien, 2012). This is especially the case for those in the asylum system. Housing international protection applicants separately from the general population (in an accommodation system known in Ireland as Direct Provision) increases the appearance of difference, and thus the likelihood of racial tension and discrimination (Hewson, 2018).

In this context, the present study set out to explore the lifetime impacts of gender-based violence on African migrant women living in Ireland, emphasizing the way that violence affects women's space for action (Kelly, 2003) and the role that intersecting stigmas play in shaping that space for action. My contention was that stigma is an element of the continuum of violence and oppression, and that, much like controlling behavior, it continues to exert a force and power over victim-survivors of violence even in the absence of direct or ongoing interpersonal violence.

The six FGDs included a diversity of participants, all first-generation immigrants to Ireland with a variety of legal statuses. As I will go on to describe, focus groups did not involve any personal disclosure, and participants were encouraged not to identify themselves as victims or survivors of violence. Participants ranged in age from 18 years to their 60s. The smallest FGD was made up of three people, the largest, 11. Two of the FGDs comprised women from Nigeria; two, women from South Sudan; and the other two FGDs were heterogeneous, including participants from Democratic Republic of Congo, Kenya, Zimbabwe, Sierra Leone, Angola, Tanzania, and Zambia. Discussions were conducted mainly in English, with some informal translation. The following section introduces the interview participants I discuss in this article.

### Four Women

The four interview participants were self-identified victim-survivors. They provided detailed testimonies of their own lives, in response to questions about their experiences of migration and violence. The harm that was outlined in interviews was experienced along a continuum of violence and oppression (Kanyeredzi, 2018), echoes of which were also present in the themes and stories that emerged in FGDs. Interview participants are introduced here, their personal details concealed through the use of pseudonyms and the removal of identifying information:

#### Shade

Shade is a Nigerian woman living in Ireland for nearly 20 years, and an Irish citizen. Her father abandoned her mother when she was young and she grew up poor and stigmatized for being in a single-parent family. She experienced sexual exploitation as a child. Her husband was physically, financially, and emotionally violent towards her for 14 years. She moved to Ireland while still in her violent marriage, with her husband travelling between Nigeria and Ireland. In Ireland, she founded her own business and achieved numerous higher level qualifications. She described numerous instances of racial discrimination in Ireland, especially in the context of work.

#### Blessings

Blessings travelled to Ireland from an East African country; at the time of the research, she was awaiting a decision on her international protection claim and was living in Direct Provision accommodation. Political violence in her country led to her mother's disappearance. She was married to a police officer who was physically and sexually violent toward her; she also experienced physical violence at the hands of other members of the police force. She said that her children experienced racism in school in Ireland, but she taught them to ignore it.

#### Maude

Like Blessings, Maude came to Ireland as an asylum seeker. She came from a country in Southern Africa and was living in Direct Provision at the time of the research. As Maude now speaks publicly about her life, I will conceal most of her story here, to maintain her anonymity. She survived IPV in her home country and Ireland, and she was also subject to sexual abuse as a child, at the hands of family friends.

#### Mary

Mary came to Ireland as part of a group of “programme refugees” from South Sudan; she is now an Irish citizen, although life in Ireland proved isolating for her. The second Sudanese Civil War began when she was a girl, and she experienced neglect and physical abuse, sexual violence, coercion at gunpoint, knife slashings, and threats to take away her child. She fled to a number of refugee camps in Uganda, where she lived for 9 years before coming to Ireland. In Ireland, she found it hard to get paid work, and felt unsupported and inhibited from pursuing her many business interests, leaving her poor, isolated, and lacking autonomy.

### Developing a Creative, Feminist Narrative Inquiry: Recruitment and Ethics

The research presented in this article involves qualitative narrative methods with women members of African migrant communities in Ireland. I began by building relationships with migrant grassroots groups and community nongovernmental organizations (NGOs). Five organizations agreed to assist me with the study, ranging in nature from a small voluntary local women's group to a national NGO representing migrant women. In each of these organizations, I found an individual who helped me recruit participants to FGDs. I spent some time getting to know these individuals and developing the project with them. FGDs were carefully constructed to be made up of small groups of women who were already known to each other and had trusting relationships.

Recruitment for IDIs occurred through the FGDs and ongoing snowball sampling. FGD participants were encouraged to get in touch with the researcher if they had a personal story they wished to share in confidence. In this way, individuals could participate in IDIs unbeknownst to the gatekeeper who facilitated their participation. Beginning recruitment with FGDs meant that the participants were not necessarily those who were highly motivated to talk about personal experiences of violence; rather, they were groups of women easily accessible to the different gatekeepers and amenable to more general discussion about women's lives and health. This proved a highly effective strategy for trust building, but it was time-consuming and ultimately yielded fewer research recruits than I had hoped. One interview participant was subsequently recruited through snowball sampling rather than an FGD.

Ethical approval for the research was granted by the ethics committee of my university in March 2017, and the study was designed with an emphasis on minimizing harm to research participants. I developed a protocol for gaining informed consent while minimizing publicity about the subject of the research, in order to protect participants from being associated with a controversial topic (Ellsberg & Heise, 2005). Participants were initially invited to an FGD to discuss “migrant women's lives and health” and put in touch with either a gatekeeper or myself (I worked with gatekeepers on the practicalities of setting up the sessions). At that point, we made it clear that the discussion would involve sensitive topics including violence, but there would be no need to disclose anything personal. I developed protocols for managing disclosures, dealing with distress, and making referrals to specialized agencies where necessary. Participant names and identifying details were changed in data transcripts and in research outputs, including this article.

Conscious that I conducted this research as an outsider to the community of African women (I am white Irish, highly educated, and relatively well-off), I practiced reflexivity through regular memoing, and created an informal mentor group of African-origin researchers to support my reflections and decisions. Reflecting on my research encounters, I noted my own intensely heightened awareness of my raced “otherness,” apparent in my relative wealth, my security of housing, my extensive social capital, and stable legal status in Ireland, and the relative ease of my life experiences. It was rare that research participants shared my discomfort with my relative privilege, but on occasion, I was challenged for choosing to research a community that was not my own. I welcomed such challenges as opportunities to discuss the purpose and possible value of the research, and also as evidence of the freedom of participants to confront, challenge, and ultimately opt out of the research encounter as they chose. As I discuss throughout this article, I dealt with my outsider status by emphasizing the “unrepeatable uniqueness” of each individual, rather than categorical similarities and differences. I also sought insight into the significance of categorical distinctions from research participants and African expert mentors whenever possible.

### Storytelling in FGDs

FGDs involved a series of discussions about social networks and violence, based on an imagined character. Here I drew on participatory and creative approaches. Some such approaches are established in research literature related to violence against women (e.g., Ellsberg & Heise 2005; Hugman et al., 2011), although the possibilities of arts-based and visual forms (e.g., Butler-Kisber & Poldma 2010; Lapum et al., 2015) are not well documented in violence against women research. Participants were invited to choose a cut-out image of a woman from a box and collectively imagine her name and life, then to discuss experiences of friendship, social networks, and violence in her life, using Venn diagrams and free listing as frameworks to gather relevant information (see Ellsberg & Heise 2005, pp. 128–153). I steered the discussion with reference to the experiences of the group and my own advance research. This use of images and story for applying these participatory tools proved surprisingly effective, and participants engaged enthusiastically with it, using it as a trigger to share stories from their own lives and those of others, weaving between fiction, celebrity stories, and personal anecdotes. The combination of moveable pictures and storytelling served to animate our imaginations, my own and those of the participants (Lapum et al., 2015). In this way, norms and attitudes related to some sensitive subjects were freely and confidently explored, while participants were actively discouraged from disclosing personal or confidential information.

The FGDs comprised groups of women who were friends or colleagues, and I prioritized creating a comfortable setting where participants would speak freely and I could observe the social norms and interpersonal dynamics that existed in networks of friendship and trust (Michell, 1999). I made extra effort, alongside gatekeepers, to create a convivial atmosphere in the FGDs, organizing them in spaces that were well known and comfortable to most of the participants (community centers, NGO offices, a private room in a public library and, in one case, in a participant's home), and providing refreshments including home baking.

#### Narrative Interviews

I conducted IDIs with the four self-identified victim-survivors in this article (introduced individually above) between June 2017 and March 2018. There was a dual axis to the interviews: I asked first about the participant's journey to Ireland; and then to tell me about the violence that they had volunteered to share. I used a semistructured narrative interview approach, allowing the interviewee as much as possible to control the narrative herself (Wigginton & Setchell, 2017). My interviewing manner was constructed so as to redress the power imbalance as much as possible, through giving participants control of the time and how it was used, using minimal encouragers and active listening techniques, and including space at the end of the interview for participants to add anything they wanted (Wigginton & Setchell, 2017, p. 258).

After the initial interview, I used cut-out images, events, and quotes to build a timeline of the overall story as I understood it, and at a follow-up interview, I invited the interviewee to reflect on the timeline and on the idea of the social impacts of violence in their lives. Following these encounters, once I had completed an initial analysis I carried out a validation workshop with participants who had taken part in FGDs or IDIs. I used the same generic cut-out images from FGDs and interviews for this workshop and was surprised when, without any instruction from me, the participants seized on the images and immediately began telling stories about the characters in them and their experiences of violence and stigma. The appeal of the approach was clear, and the narratives that workshop participants created independently closely mirrored the themes that I was validating.

#### Narrative Analysis

The use of narrative extended to my analytical approach. FGDs and IDIs were coded thematically according to a modification of grounded theory (Gioia et al., 2012), but IDIs were additionally studied to trace the overall narrative arcs and central plots described by interviewees themselves (Floersch et al., 2010). Studying the individual interviews, I found that the underlying plot of very different interview transcripts shared a common trajectory from suffering to “spectacular overcoming” (Taylor, 2018). There was scope within the interview structure for any number of temporal and causal tellings. However, in the cases of Shade, Blessings, and Mary, the overall plot told a similar story of violence, followed by escape, followed by struggle and often poverty, ending in the expression of personal pride or satisfaction at “having overcome,” with an emphasis on individual agency and the brightness of the future. In the three cases I have named, this narrative demonstrated a personal narrative identity of “resilient survivorship” (Taylor, 2018), which was closely mirrored in the themes that emerged from FGDs. Hill Collins (2000), the Black feminist sociologist, describes how African American women adopt personas of strength or resilience in order to withstand the daily adversities of life in a racist society. In my study, the levels of identification with the “strong Black woman” (Beaubeouf-LaFontant, 2008; Kanyeredzi, 2018) gave this persona the nature of a controlling image (Hill Collins, 2000), which defined the possibilities for narrative identity construction in the aftermath of violence. The underlying narrative identity of survivorship and overcoming became very important for the subsequent research analysis, as I shall outline in the following section.

#### Turning Narratives into Things: The Gift of Books

**Figure 1.**
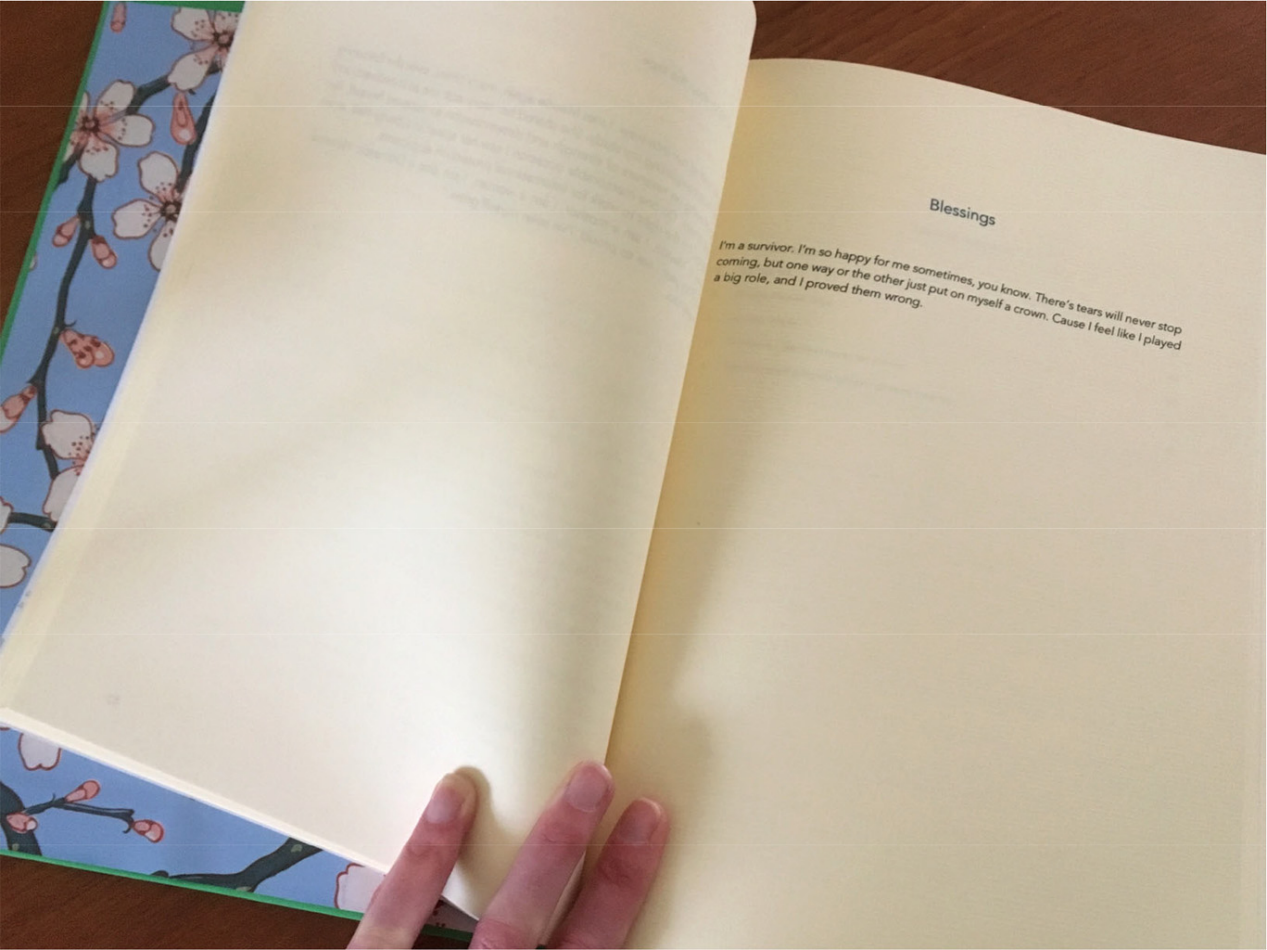
Handmade book of interview transcript.

**Figure 2.**
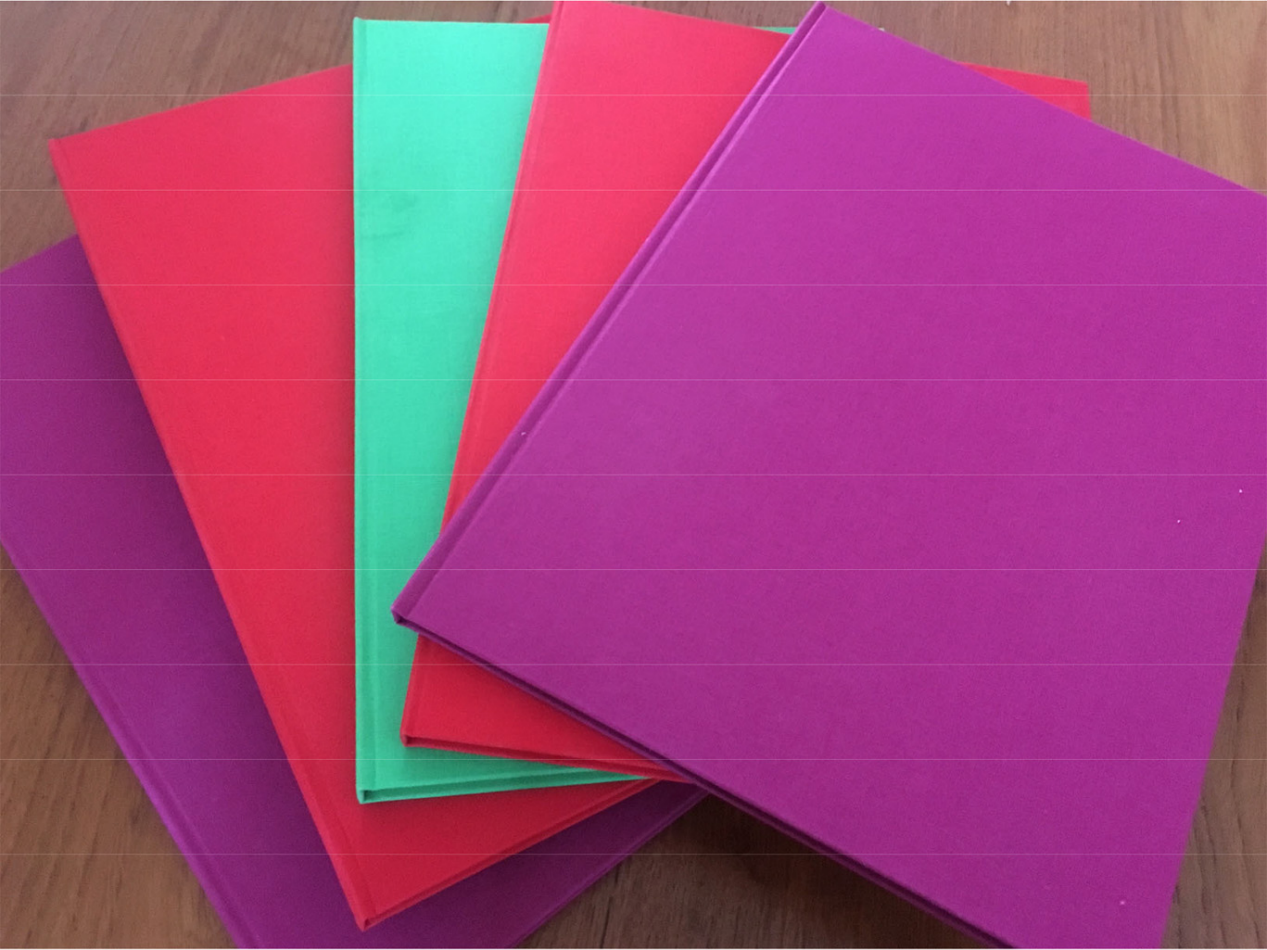
Handmade books.

Once the fieldwork was complete, I worked with an artist friend, Íde Ward, to produce the interview transcripts as individual hand-bound hardback books, and returned them to each of the participants. These books were unique single editions, suggested in the first instance by specific comments from two participants. Both Maude and Shade had a strong sense of their own survival as exemplary and potentially instructive, and both told me they hoped someday to write books of their own. Returning their transcripts to them as something beautiful and unique thus served the purpose of acknowledging their authorship and giving it back to them, in some way symbolically sharing the power that their knowledge and narratives might hold. I did this mindful of the observation that often one's narrative of trauma must be witnessed “for survival as an autonomous self to be complete” (Brison, 2002), and equally mindful of Imogen Tyler's exploitative and exclusionary research experience which opened this article (Figures 1 and 2).

#### Navigating Stigma and Violence

The study set out to investigate the ways in which victim-survivors navigated stigma in the aftermath of life experiences of violence. It found that research participants navigated stigma in three primary ways: through relationship management, maintaining appearances, and strategic telling and hiding of their violent histories.

Relationship management was a constant act of assessing one's standing with different people. In both FGDs and interviews, participants explained that in the aftermath of violence, women would be careful about the people they allowed in their circles. Shade was most explicit about this, stating:

Sometimes you just have to kick the Nigerians away.

Although her diaspora community was immensely important to Shade, she expressed her discomfort at the awareness that she was often being judged as a single mother and divorcee. Knowing this damaged her trust with her Nigerian community, although it did not help with the difficult task of building relationships with Irish people.

Maintaining appearances arose in both FGDs and interviews as a core skill of victim-survivors of violence. It was perhaps most eloquently explained in FGD A, when one participant described how a fictional survivor of domestic violence in a vignette would present herself in public:

you may see her [the fictional woman] with fake stuff shining with make-up and everything smiling, that's what they call, Suffering and Smiling. (S/FGD A)

The final approach to navigating stigma which I documented was in strategic telling and strategic hiding of histories of violence. This was relevant both to the research findings, and to the methods used to do the research, and is discussed further in the following section.

## Discussion: The Promise and Limits of Narrative

Stigma essentializes individuals, devaluing their whole identity (Goffman, 1963) and emphasizing a single label, attribute, or characteristic. Narrative research methods promise to restore agency and uniqueness—in the best possible scenario, empowering the research participant (Eastmond, 2007) and draining the research discourse of stigmatizing content (Wigginton & Setchell, 2016). In this section, I show how the study had the intended effects of limiting stigma in research settings by maximizing the agency of the participants. I explore the connection between narrative for research purposes and as it is used at other points in the participants' lives. And I look at the ways that narrative can be a tool of stigma, and how this played out in the research study.

This study demonstrated the value of narrative methods, deployed creatively, for eliciting rich personal reflection on stigmatized topics in violence against women research. Participants were enthusiastic about the creative narrative tools used, whether in FGDs, interviews, or in the ongoing back-and-forth communication of my research; they responded particularly well to the use of images, tactile materials, and the presentation of interview testimonies in bound-book form. In spite of being an outsider in many ways to the groups studied, narrative approaches functioned to emphasize the “unrepeatable uniqueness” (Cavarero, 2000) of each individual research participant, thus downplaying the categorical differences that might separate me from the group.

The intrinsic value of narrative approaches for victim-survivors in research were expressed by most of the interview participants. For example, Blessings said, of coming to my house and doing an interview:

It's not easy you know to talk about such things. But then, it also give me power. Yeah, it also give me power. And it also shows me that I did not deserve all this. You know. What I went through. (Blessings)

In this way, Blessings clearly related the act of telling her story to her own self-care. For her, testimony connected to processes of recovery and transformation, and was therapeutic in a way (Brison, 2002; Eastmond, 2007; Fontes, 2004).

These interview participants (with the exception of Mary) informally expressed a belief to me that sharing individual narratives with a researcher might serve to help others apart from themselves; that is, they had an instrumental value. Recruitment for IDIs in this study proved difficult, so that those who did agree to give interviews were clearly motivated to do so. The interview participants were all somewhat practiced in narrating parts of their personal histories. Their reflections on testimony, narrative, and the instrumental value of their stories thus extend far beyond the research encounter and provide useful insight into some meanings of narrative for victim-survivors in research. Shade most clearly expressed a belief in the power of her personal narrative:

I plan to one day, when I’m ready, I plan to share my story because I don't want to hide it, I want to ... I want somebody to be inspired, right? (Shade)

Maude too reflected on how she had drawn on her own experience to provide succor to other women in similar contexts:

And every chance I get, if someone is talking about the subject [of domestic violence], I talk about my own experience. I don't go into details but I tell them, “You know what? You can walk out. And make it. Because the most important person is You.” (Maude)

Here, Maude is drawing on her own experience to help others, while simultaneously withholding her story (“I don't go into details”), thus protecting herself from the potentially harmful impacts of disclosure, which include (though they are not limited to) stigmatization.

Blessings, the least publicly engaged of the four interview participants, described a unique and important moment of exploring the power of story. Towards the end of her interview, she expressed a keen desire to reach out to other victim-survivors:

I wish I could help some people. You know, there was this time where I wanted to open a group on Facebook, for people that they’re going through abuse or are the victims. Just months ago before I met you.

That thing, you know, I said I’m so powerful. Thinking of such huge step. Yeah. I pretty powerful.

But then no, I didn't do it, but I know one day. One step at a time. You know. I wish I could, help more people. (Blessings)

Having pulled herself back from that public act of sharing, Blessings believed that talking to me was an alternative way of safely using her story for the benefit of others. There is an evident tension between sharing and self-protection which Shade explained, demonstrating a keen awareness of the risk of gossip and stigma, even among potential allies:

I think if somebody says something that triggers ... someone may be sharing their experience and it's like I *connect*, because I *know* ... but I don't … with a lot of women, I don't go about telling them my own story unless, you know, I could touch on it for lots of reasons I could tell them yes, but I don't go deep and discussing. … (Shade)

All of these reflections demonstrate in different ways that narrative is a practice that is alive in the participants' lives well beyond the research encounter, and that the participants had given thought to the impacts of telling their stories, for themselves and others. In their everyday lives, Maude, Blessings, and Shade moved between strategic speaking and strategic silences (Kanyeredzi, 2018), protecting themselves from external judgment by offering a listening ear to others while revealing just the right amount about themselves. Methodologically, then, the interview served as a liminal space in which the self-conscious participant could explore and test the possibilities of narrative without being directly interrupted by stigmatization or other risks. There are inevitable limits to this possibility, which I explore in more detail further on in this discussion.

By returning the interview transcripts to the participants as beautifully bound books, I was consciously validating the status of the research participants as the authors of their own narratives. The hard-bound books, although they were completely one-off objects, were intended to represent symbolically the public legitimacy of the individual testimonies, and perhaps to encourage participants to continue experimenting cautiously with storytelling. For Maude and Shade, who had both mentioned the desire to share their stories publicly, I thought it would be helpful for them to consider what their words looked like in print, and the feelings this evoked in them—gently pushing the liminal space a little further. Both responded positively and said again that they would like to share their own stories publicly, eventually. In this way, I tried to validate participants' narratives by returning to them in the form of a physical artifact, a representation of their story being witnessed (Brison, 2002).

One result of this participant-centered, creative, and narrative approach to both FGDs and IDIs was that stigma and violence were given equal space alongside the many other issues that confronted research participants. The narratives generated were not *about* violence or stigma in and of themselves, but rather about accommodating those things, living alongside them. This slantwise approach to information proves useful for research on sensitive topics. Mary's narrative was dominated neither by violence nor exclusion, but rather by the overwhelming impact that bureaucracy had had on her life, principally in large-camps run by the United Nations High Commission for Refugees (UNHCR) in Uganda, and subsequently in Ireland. FGD C was dominated by the theme of loneliness and isolation for South Sudanese refugee women living in Ireland. Incidents of stigma and violence were revealed without being centered. Narrative thus provided for richer and more nuanced data than a more direct methodology and, in turn, demanded a more considered analytical approach, the outcomes of which I discuss below.

### The Limits of Narrative Inquiry

We have seen how narrative methods can create space for creative and ethical approaches to researching stigmatized topics and can generate rich and valuable qualitative data. Nonetheless, it is vital to note that narratives, even those given freely and directed by the participant, are also constrained and shaped by all manner of external forces: They are co-constructed in the interview space and most especially in the focus group situation, and they are socially constrained in all contexts.

Especially relevant to this study, narrative is a central tool in the production and reproduction of stigmatizing beliefs. Cultural settings provide their members with the tools and scripts for doing narrative: “a limited stock of possible story-lines,” as Polletta (1998, p. 424) describes it, at the same time rendering certain stories unthinkable and untellable. It is thus naive to imagine that just because an individual or group is granted the space to tell “their own” story, that the story is free of external constraints. Canonical narratives shape not only what we say, but what we imagine. In the case of the South Sudanese refugee women interviewed by Tankink (2013), sexual violence fell so far outside the realm of available narratives (Tankink uses the term “cultural master narratives”), that to disclose such violence was to endanger the individual in her group. In this case, the risk was directly related to the stigma of talking about sexual violence, which would result in the exclusion of the victim-survivor, an unthinkable outcome given the importance research participants attached to their small network of co-ethnic refugees.

The canonical narrative which haunted this research study was that of the “resilient survivor,” suffering violence stoically in order to emerge triumphant and strong. African American victim-survivors of violence against women have been found to identify strongly with the persona of the strong Black woman (Potter, 2007), characterized as stoic, uncomplaining, and resilient. A comparable identification is seen among African and Caribbean-origin women in the United Kingdom (Kanyeredzi, 2018). In the analysis of overarching narrative plots, three of the four participants represented here (with the exception of Maude) presented stories that followed a similar trajectory, despite recounting very different backgrounds, contexts, and experiences. There was scope within the interview structure for any number of temporal and causal tellings, but in the cases of Shade, Blessings, and Mary, the overall plot tells a story of violence followed by escape, characterized at all times by struggle and often poverty, but ending in the expression of personal pride or satisfaction at “having overcome,” with an emphasis on individual agency and the brightness of the future.

Shade encapsulated this storyline most clearly when she said of the difficulties she experienced after leaving her husband:

I could go hungry, I just ... I want my freedom. (Shade)

Polletta (2009) describes this telling as a “familiar escape tale,” one of the very few storylines available to so-called battered women. She argues that this is problematic because it is unrealistic; I suggest that it is chosen by victim-survivors in a context of stigmatization of most of the alternatives. Self-description as a “victim” would imply highly stigmatized weakness or dependency, since racialized women are often negatively stereotyped in their own communities and in dominant white society for perceived dependency (Hill Collins, 2000). Self-description as an independent, autonomous agent whose response to violence was rational and deliberate would invoke the many stigmas against transgressional femininity (e.g., labelling of single parents, bad mothers, welfare queens). A personal identity that encompassed lasting harm or residual anger is similarly stigmatized as unfeminine. Thus, escape and survival were effectively the storyline that most interview participants and many FGD participants reached for (see also Kanyeredzi, 2018).

When I returned the transcripts to participants in the form of books, I was not just validating their status as the authors of their own experience, but also reinscribing the controlling image of the strong Black woman (Kanyeredzi, 2018; Potter, 2007). I wanted to challenge participants' statements about their own strength, to encourage them to voice other possible drivers of their plots, such as anger, powerlessness, or resistance, but I was methodologically and ethically committed to allow them to lead the reflection. This is a challenge of the socially constructed narrative, whereby the stigmas written into the social context of the research encounter are replicated in the data. I was encouraged, then, by a dynamic that arose incidentally in the final group activity I conducted for the research.

The resilient survivor narrative dominated FGDs just as it did IDIs, but it did not go entirely unchallenged. In the validation workshop, two out of three participants enthusiastically (and with no direction to that effect from me, the facilitator) replicated stories of an imagined resilient survivor, spectacularly overcoming her experience of violence. What was interesting in this case was that Maude, the third participant, actively challenged this canonical narrative, which stigmatized weakness or dependency. At the workshop, a participant I called Y expressed solidarity with the abused woman she had imagined in her vignette, endorsing her character's resilient survivor identity:

We can't cry. We have to overcome the storm. We can't show our vulnerability. We have to be strong in our moment of tears. (Y, Validation workshop)

Maude replied with a very different perspective. She said, of a vignette that she had created around a character she named Linda:

You are taught you always have to be strong; Linda doesn't always agree with that. You need to go through the emotions as and when they come to get strong. You need to give yourself a chance to heal. (Maude, Validation workshop)

This was a direct challenge to the negative labelling of showing weakness or vulnerability which arose regularly in both interviews and FGDs. The exchange demonstrates the potential for counter-narratives to emerge and be explored safely, without their proponents bringing stigma directly on themselves. The third person device of talking about a fictional character meant that no personal experience was directly judged or assessed, and no individual was directly stigmatized, though stigmatizing beliefs could be voiced and explored and even contradicted. Arguably the attention paid to creating welcoming and safe spaces also allowed for such counter-narratives to be explored safely. Tankink (2013) concludes her paper on the silence of South Sudanese women with the reflection that South Sudanese women love to tell stories. She argues strongly in favor of respecting the silences of refugee women with regard to sexual violence. She further suggests that the telling of non-personal stories can help women to “express what needs to be expressed without explicitly describing an event or experience” (p. 401). This is precisely what happened in the exchange between Maude and Y above, although the stigma attached to female vulnerability is certainly weaker than the stigma of sexual violence. While they were discussing the vignettes they had created, there was a deeper conversation about narrative possibilities, in this case opening a space for stigmatized narratives of weakness.

## Conclusion

With this article, I have set out to explore the meanings of narrative in research on the stigmatized subject of violence against women, with participants who are subject to multiple additional intersecting stigmas (racism, patriarchal stigma, welfare stigma, and mental health stigma, in particular). I have described the creative and participatory methods that I used in a qualitative study involving IDIs and FGDs with African migrant women living in Ireland. An impact of my PhD study which I had not anticipated was that I fell in love with narrative research, its methods, and theory alike, and in this article I demonstrate how it can be used for effective and probing feminist research.

There are three main messages from this article for using narrative to study violence against women and its associated stigmas. The high ethical standards required of researchers of violence against women are well established, and are a minimum prerequisite for all research on the topic (see Ellsberg & Heise, 2005; Fontes, 2004). Assuming such standards are met, this article presents further reflections on the uses of narrative. First, group discussions can be used to good effect to explore and expand narrative possibilities. As well as meeting high ethical standards, I found it helpful to set up welcoming, friendly, and fun spaces (I note that in our current pandemic times, this is especially challenging). A key feature was the creative use of third person story-telling to facilitate narrative discussion without the risk of disclosure or exposure; such techniques are highly recommended for gender-based violence research with refugee communities. They provide the freedom to name issues without individual exposure, and they may even afford the possibility—as they did in this study—for research participants to challenge dominant and often stigmatizing narratives with alternative tellings.

Second, for individual interviews, it is noted that recruitment is extremely challenging and the barriers to racialized migrant women sharing personal experiences of violence in research are high. Those who do choose to participate are therefore likely to be highly motivated to do so (see also Kanyeredzi, 2018), and narrative interviews are appropriate for such highly motivated participants, giving them autonomy to direct the telling. In this article, I have shown how interview participants experimented with public testimony, but engaged frequently in strategic silences, owing to the risks of stigmatization within their communities. They actively used the research interview to explore the possibilities of testimony and narrative, and I gently encouraged this in the act of returning their transcript to them as a personal and beautiful book. Concrete creative interventions of this sort can help to bridge the space between confidential interviews and the wider potential of narrative testimony for change, and allow the participant to explore it.

Finally, it is necessary to remember at all times that as long as stigmatizing beliefs are present in wider society, they will be present in the research encounter, however it is structured. The astute researcher must be alert to this fact and bear it in mind when facilitating discussions and when analyzing data. It is not possible to eliminate stigmatizing messages, but it is essential to recognize them and not to casually replicate them by reporting them uncritically.

Stigma presents a huge challenge to qualitative research on violence against women. This must be acknowledged because it cannot be wholly overcome. Narrative is an ideal way of contesting stigma, although the stigma encoded within narratives must give reason for caution. This can best be done, first, by using creative means to develop third-person storytelling to play with existing narratives in group settings (and, indeed, in individual ones). Second, narrative should be acknowledged as an ongoing process, and connections can be made between the research narrative and other opportunities. One way I did this was through returning transcripts as a gift book. And finally, in facilitating and analyzing such research, a high level of awareness of stigmatizing beliefs should always be maintained, as they can so easily become unintentionally replicated.

It is possible to set up research encounters in such a way that individuals are protected from being directly labelled themselves, and where they have maximum facility to safely explore alternative tellings and challenge stigmatizing beliefs. The participatory and creative narrative approaches I describe here represent a promising approach to this end.
